# Prevalence of selected cardiometabolic risk factors in the global ART-naïve HIV infected population: A protocol for a systematic review and meta-analysis

**DOI:** 10.1371/journal.pone.0286789

**Published:** 2023-06-08

**Authors:** Peter Vanes Ebasone, Nasheeta Peer, Anastase Dzudie, Andre Pascal Kengne

**Affiliations:** 1 Faculty of Health Sciences, Department of Medicine, University of Cape Town, Western Cape, South Africa; 2 Clinical Research Education, Networking & Consultancy (CRENC), Douala, Cameroon; 3 Non-communicable Disease Research Unit, South African Medical Research Council, Cape Town, South Africa; 4 Faculty of Medicine and Biomedical Sciences, University of Yaounde 1, Yaounde, Cameroon; Università degli Studi di Milano, ITALY

## Abstract

**Introduction:**

People living with HIV/AIDS (PLHIV) are at increased risk of cardiometabolic diseases attributable to the effects of the virus, antiretroviral therapy (ART) and traditional risk factors. Most studies have focused on assessing the effect of ART on cardiometabolic diseases in PLHIV with fewer studies assessing the cardiometabolic risk profile prior to exposure to ART. Therefore, this protocol is for a systematic review and meta-analysis to estimate the global prevalence of selected cardiometabolic risk factors in ART-naïve PLHIV and their association with HIV specific factors.

**Methods:**

We shall conduct a systematic search of observational studies on the prevalence of obesity, hypertension, diabetes, and dyslipidaemia in ART-naïve PLHIV and their association with HIV specific characteristics. We will search PubMed-MEDLINE, CINAHL, SCOPUS, Academic Search Premier, Africa-Wide Information and Africa Journals Online databases to identify relevant studies published before June 2022. Two authors will independently screen, select studies, extract data, and conduct risk of bias assessments. Disagreements between the two authors will be resolved by consensus or consulting a third reviewer. Data consistently reported across studies will be pooled using random-effects meta-analysis. Heterogeneity will be evaluated using Cochrane’s Q statistic and quantified using I^2^ statistics. The Preferred Reporting Items for Systematic reviews and Meta-Analysis protocols (PRISMA-P) 2015 guidelines are used for the reporting of this protocol.

**Discussion:**

This review will help determine the burden of selected cardiometabolic diseases in ART-naïve HIV-infected populations and the contribution of HIV infection, independent of ART, to cardiometabolic diseases in PLHIV. It will provide new information that can help orientate future research and potentially guide healthcare policy making. This is part of a thesis that will be submitted to the Faculty of Health Sciences, University of Cape Town, for the award of a PhD in Medicine with protocol ethical clearance number (UCT HREC 350/2021).

**Registration:**

PROSPERO: CRD42021226001. https://www.crd.york.ac.uk/prospero/display_record.php?ID=CRD42021226001.

## Introduction

About 38.4 million people globally are infected with the Human Immunodeficiency Virus (HIV), most of whom reside in Sub-Saharan Africa (SSA) [1]. The incidence of HIV remains high with 1.5 million new infections in 2021 alone [1]. The introduction of potent antiretroviral therapy (ART) has led to a dramatic improvement in the quality of life with decreased morbidity, and longevity of people living with HIV/AIDS (PLHIV). As more PLHIV are now living beyond 50 years of age [2,3], they are more exposed to traditional risk factors such as ageing and a genetic predisposition together with lifestyle behaviours associated with unhealthy diets, physical inactivity, smoking and alcohol misuse. Alongside the high burden of HIV infection worldwide, there is a growing non-communicable diseases (NCDs) epidemic. These NCDs include cardiometabolic diseases such as obesity, hypertension, type 2 diabetes mellitus (hereafter referred to as diabetes) and dyslipidaemia [4].

Some studies have shown that cardiometabolic disease risk is higher in PLHIV on ART than those who are ART-naïve and higher among HIV positive people compared to those who are HIV negative [5–7]. This is due to the effect of the virus itself, related opportunistic infections, and ART [8,9]. The HIV virus accelerates and accentuates cardiometabolic changes by decreasing the body’s regenerative ability, hence expediting the process of immunosenescence and systemic ageing [10–12]. It also induces chronic inflammation, sustained immune activation and increases the risk of thrombosis [13,14]. The virus directly causes lipid abnormalities including high triglycerides and low high-density lipoprotein cholesterol (HDL-C) [15,16] and is a strong determinant of myocardial infarction, stroke, and diabetes [17–19]. On the other hand, ART regimens, such as protease inhibitors have been shown to be associated with increased levels of serum low-density lipoprotein cholesterol (LDL-C), triglycerides (TG), total cholesterol (TC) and insulin resistance [8,19,20]. The new WHO recommended first line regimen Dolutegravir have been shown by early studies to be associated with significant increased body weight and an increased incidence of obesity [21,22].

Most studies have focused on the prevalence of cardiometabolic risk factors in PLHIV on ART. Fewer studies have explored cardiometabolic diseases in the ART-naïve HIV infected population. Particularly, the global prevalence of these cardiometabolic risk factors in the ART-naïve population is unknown. Assessing this prevalence will help inform and guide future research and public health interventions. Therefore, this study aims to assess the prevalence of obesity, hypertension, diabetes, and dyslipidaemia (high TC, high LDL-C, high TG, low HDL-C) in the global HIV infected ART-naïve population; and the association of HIV specific factors with these cardiometabolic risk factors.

### Review questions

1. What is the prevalence of obesity, hypertension, diabetes, and dyslipidaemia (high TC, high LDL-C, high TG, low HDL-C) in the global ART-naïve HIV population?
2. What is the association between HIV specific factors including time since diagnosis, severity of the disease, CD4 count and/or viral load and these selected cardiometabolic risk factors?

## Materials and methods

### Eligibility criteria

#### Inclusion criteria

Studies involving human subjects and fulfilling the following criteria will be included:

1. **Study designs**: cross-sectional, case–control and cohort studies.
2. **Study participants**: ART-naïve patients (aged 15 years or above), living with HIV/AIDS worldwide.
3. **Clinical outcomes:** the prevalence of any of the following: obesity, hypertension, diabetes, or dyslipidaemia (high TC, high LDL-C, high TG, low HDL-C) or sufficient data for estimating the prevalence, with/without HIV specific factors (time since diagnosis, WHO stage, viral load and/or CD4 count).
4. **Time-period**: we will consider all published and unpublished data available up to the time of the review.
5. **Study settings**: health facilities or community-based settings.
6. **Language**: all studies reported in the English or French languages.
7. For studies that reported data from the same study population, the study with the largest number of participants will be included.

#### Exclusion criteria

Studies with the following characteristics will be excluded:

1. Studies lacking prevalence measurements or sufficient data to perform these estimates even after contacting the primary investigators.
2. Case series, case reports, reviews, clinical trials, commentaries, and editorials.
3. Studies not performed in human participants or published in languages other than English and French.

#### Sources of information

The systematic review’s methods are reported according to the Preferred Reporting Items for Systematic reviews and Meta-Analysis protocols (PRISMA-P) 2015 Guidelines (S1 Checklist) [23]. This review protocol has been registered at the international prospective register of systematic review and meta-analysis **(**PROSPERO: CRD42021226001**)**.

### Search strategy for identifying studies

#### Electronic searches

We will undertake a comprehensive electronic search across PubMed-MEDLINE, CINAHL, SCOPUS, Academic Search Premier, Africa-Wide Information and Africa Journals Online databases to identify relevant studies. The search shall be conducted using a predefined comprehensive and sensitive search strategy that will comprise combinations of MESH terms, CINAHL headings, and free words relating to cardiometabolic risk factors, ART-naïve and HIV/AIDS. We will use controlled vocabularies synonyms to identify related terms. The search strategy was reviewed following the PRESS Peer Review of Electronic Search Strategies Guidelines by an Information Specialist [24]. S1 File contains the detailed search strategies for PubMed-MEDLINE, SCOPUS and CINAHL. We will update the search prior to publication to include any additional eligible papers published recently.

#### Grey literature

We will search reference lists of relevant citations and scan the reference lists of review papers and conference proceedings. We will also examine publications on the websites of key organisations such as UNAIDS, WHO, and the International AIDS Society. Key experts in the field will be contacted for any unpublished study.

### Study records

#### Data management

Search results shall be imported into EPPI-Reviewer software for de-duplication. Well-structured and standardized questions developed in accordance with the inclusion criteria shall be used for screening studies in EPPI-Reviewer. EPPI-Reviewer is a web-based software program for screening, managing and analysing data in systematic reviews and meta-analysis [25].

#### Screening

Two investigators will independently screen titles and abstracts using the inclusion criteria. Full texts will then be obtained and screened using a standardised and pre-tested form to include eligible studies. Disagreements will be resolved by consensus or by consulting a third investigator. We will contact corresponding authors of potentially eligible studies for relevant data that were not reported. Reasons for exclusion of non-eligible studies will be documented. The entire selection process will be summarised in a PRISMA flow chart (S2 File).

#### Data extraction

The data for this review will be extracted using a purposeful design and piloted extraction form on Excel. Two investigators will independently extract data from included studies and any inconsistencies or disagreement shall be resolved by consensus or consultation with the third investigator.

#### Data items

The data to be extracted will include 1) Author and paper details [first author name, year of publication, language of publication]; 2) Study characteristics [country, study design, coverage (national or sub national), study setting (urban vs rural or both), study period, sampling method, participants age limit, sample size, response rate]; 3) Participants’ characteristics [mean or median age and age range, gender (proportion of males), employment status, level of education, lifestyle habits (smoking, alcohol misuse), 4) HIV-related factors (time since diagnosis, severity of the disease, CD4 count and/or viral load, mean/median CD4, proportion with CD4 count <200, median duration of HIV); and 5) Prevalence measures [number of participants with CMRFs of interest and definition of each CMRF]. 6) Association of HIV specific factors and CMRFs (measure of association, effect size estimate, standard error of effect size and adjustment for confounders).

#### Assessment of methodological quality and risk of bias

Two reviewers will independently score the quality of the included studies. For each study, the risk of bias and evaluation of methodological quality will be checked using the risk of bias tool for prevalent studies checklist adapted from Hoy et al [26]. The overall risk of bias will be scored and summarised as low, moderate, or high risk (S2 Checklist). Any discrepancies will be resolved by consensus or by consulting the third investigator. Inter-rater agreement on screening, data extraction and methodological quality will be assessed using Cohen’s κ coefficient [27]. The quality assessment scores will be presented in a table for easy interpretation.

#### Data synthesis, analysis, and assessment of heterogeneity

A summary of prevalence data will be presented by country and continent. Meta-analysis and meta-regression analysis will be conducted for identical variables across studies. The study-specific estimates will be pooled through a random-effects model, to obtain the overall summary estimate of the prevalence and associations across studies, after stabilising the variance of individual studies with the use of Freeman-Tukey double arc-sine transformation [28]. This transformation will help reduce the effect of extremely high or extremely low measurement values on the pooled estimate. Heterogeneity will be evaluated by the Cochrane’s Q statistic and I^2^. I^2^ values of 25%, 50% and 75% will be deemed to represent low, medium, and high heterogeneity, respectively. Funnel plots together with the Egger test of bias will be used to investigate the publication bias [29].

We will also conduct subgroup and meta-regression analyses to compare the estimates across major predictive characteristics and assess the consistency of the effects across those subgroups. Major grouping characteristics will include gender, age group, geographic region, time the study was published, sample size, severity of disease, study design, etc. We will report the total number of relevant factors investigated across all studies, and for each factor, the number of times it was reported to be associated with the outcome. We will further report on the measure of association used for each factor across studies, with indication of whether those measures were adjusted for confounders or not.

Data analyses will use the ‘*meta*’ package of the latest version of R statistical software (R Foundation for statistical computing, Vienna, Austria).

#### Sensitivity analysis

We will use the leave-one-out jackknife sensitivity analysis in which one primary study is excluded at a time. This will help us assess if specific studies are influencing the overall estimates. We will then compare the new pooled prevalence or effect size estimate with that of the original one.

## Discussion

Since cardiometabolic diseases in PLHIV are driven by HIV infection per se and ARTs, together with traditional risk factors, it is important to understand the differential contribution and distribution of cardiometabolic risk factors among the HIV-infected who are not yet exposed to ARTs. This review will help determine the burden of selected cardiometabolic diseases in ART-naïve HIV-infected populations and the contribution of HIV infection, independent of ART, to cardiometabolic diseases in PLHIV. Since most published studies have largely focused on measuring the prevalence of cardiometabolic diseases and the associated impact of ARTs in PLHIV, it is important to estimate this prevalence in the ART-naïve PLHIV worldwide. This study therefore aims to provides new information that can help orientate future research and potentially guide healthcare policy making. If a high prevalence of cardiometabolic disease among ART-naïve PLHIV is demonstrated, this may indicate an increased need to strengthen efforts for earlier screening, diagnosis and treatment of cardiometabolic diseases in PLHIV.

The selection of studies published in the English or French languages only may prevent relevant data from studies published in other languages from being included. This review will, however, identify gaps in the current literature on this topic and provide direction for future research in ART-naïve PLHIV.

### Reporting of this review

The proposed systematic review will be reported following the PRISMA guidelines [30]. We intend to publish a PRISMA checklist alongside the final report.

### Potential amendments

We do not intend to make any amendments to the protocol, to avoid the possibility of outcome reporting bias. However, any amendments that do prove necessary will be documented and reported transparently.

## Supporting information

S1 Checklist PRISMA-P 2015 checklist. (DOCX)

S2 Checklist Quality assessment checklist for prevalence studies. (DOCX)

S1 File Search strategy for PubMed-MEDLINE, SCOPUS, CINAHL. (DOCX)

S2 File PRISMA 2020 flow diagram for new systematic reviews. (DOCX)
