# Supplementary material for: Prevalence of selected cardiometabolic risk factors in the global ART-naïve HIV infected population: A protocol for a systematic review and meta-analysis
Source: PLoS One. 2023 Jun 8;18(6):e0286789. doi: 10.1371/journal.pone.0286789 (PMC10249803; doi:10.1371/journal.pone.0286789)
Supplement: S2 Checklist — (DOCX) [file pone.0286789.s002.docx]

S2 Checklist. Quality assessment checklist for prevalence studies (adapted from Hoy et al [1])

| Name of author(s): |  |  |
| --- | --- | --- |
| Year of publication: |  |  |
| Study title: |  |  |
| **Risk of bias items** | **Risk of bias levels** | **Points**  **scored** |
| 1. Was the study’s target population a close representation of the national population in relation to relevant  variables, e.g. age, sex? | **Yes** (**LOW RISK**): The study’s target population was a close representation of the national population. | 0 |
|  | **No** (**HIGH RISK**): The study’s target population was clearly NOT  representative of the national population. | 1 |
| 2. Was the sampling frame a true or close representation of the target population? | **Yes** (**LOW RISK**): The sampling frame was a true or close  representation of the target population. | 0 |
|  | **No** (**HIGH RISK**): The sampling frame was NOT a true or close  representation of the target population. | 1 |
| 3. Was some form of random selection used to select the sample, OR, was a census undertaken? | **Yes** (**LOW RISK**): A census was undertaken, OR, some form of random selection was used to select the sample (e.g. simple random sampling,  stratified random sampling, cluster sampling, systematic sampling). | 0 |
|  | **No** (**HIGH RISK**): A census was NOT undertaken, AND some form of  random selection was NOT used to select the sample. | 1 |
| 4. Was the likelihood of non-response bias minimal? | **Yes** (**LOW RISK**): The response rate for the study was ≥75%, OR, an analysis was performed that showed no significant difference in relevant  demographic characteristics between responders and non- responders | 0 |
|  | **No** (**HIGH RISK**): The response rate was <75%, and if any analysis comparing responders and non-responders was done, it showed a significant difference in relevant demographic characteristics between  responders and non-responders | 1 |
| 5. Were data collected directly from the  subjects (as opposed to a medical records)? | **Yes** (**LOW RISK**): All data were collected directly from the subjects. | 0 |
|  | **No** (**HIGH RISK**): In some instances, data were collected from a proxy or medical records. | 1 |
| 6. Were acceptable case definitions  used in the study? | **Yes** (**LOW RISK**): Acceptable case definitions were used. | 0 |
|  | **No** (**HIGH RISK**): Acceptable case definitions were NOT used | 1 |
| 7. Were reliable and accepted diagnostic methods for diagnosing cardiometabolic risk factors of interest utilised? | **Yes** (**LOW RISK**): Valid and reliable and accepted diagnostic methods were used (if this was necessary) | 0 |
|  | **No** (**HIGH RISK**): Diagnostic methods had NOT been shown to be  reliable or valid (if this was necessary). | 1 |
| 8. Was the same mode of data collection used for all subjects? | **Yes** (**LOW RISK**): The same mode of data collection was used for all  subjects. | 0 |
|  | **No** (**HIGH RISK**): The same mode of data collection was NOT used  for all subjects. | 1 |
| 9. Were the numerator(s) and denominator(s) for the calculation of the cardiometabolic risk factors of interest appropriate? | **Yes** (**LOW RISK**): The paper presented appropriate numerator(s) AND denominator(s) for the parameter of interest (e.g. the prevalence of diabetes). | 0 |
|  | **No** (**HIGH RISK**): The paper did present numerator(s) AND denominator(s) for the parameter of interest but one or more of these  were inappropriate. | 1 |
| 10. Summary on the overall risk of study bias | **LOW RISK** | 0-3 |
|  | **MODERATE RISK** | 4-6 |
|  | **HIGH RISK** | 7-9 |

1. Hoy D, Brooks P, Woolf A, Blyth F, March L, Bain C, et al. Assessing risk of bias in prevalence studies: modification of an existing tool and evidence of interrater agreement. J Clin Epidemiol. 2012;65: 934-939.
