# Supplementary material for: Prevalence of selected cardiometabolic risk factors in the global ART-naïve HIV infected population: A protocol for a systematic review and meta-analysis
Source: PLoS One. 2023 Jun 8;18(6):e0286789. doi: 10.1371/journal.pone.0286789 (PMC10249803; doi:10.1371/journal.pone.0286789)
Supplement: S1 File — (DOCX) [file pone.0286789.s003.docx]

**S1 File: search strategies for the study titled prevalence of selected cardiometabolic risk factors in the global ART-naïve HIV infected adult population: a protocol for a systematic review and meta-analysis**

Databases: PubMed-MEDLINE, SCOPUS, CINAHL

**PubMed-MEDLINE**

Search Date: 02/06/2022

| **Search** | **Search Terms** | **Hits** |
| --- | --- | --- |
| 1 | Cardiometabolic diseases[tw] OR Cardiometabolic disease[tw] OR Cardiometabolic syndrome[tw] OR Cardiometabolic traits[tw] OR Cardiometabolic risk factors[tw] OR Cardiometabolic factors[tw] OR cardiovascular[tw] OR Metabolic Syndrome[tw] OR Metabolic Syndrome X[MeSH Terms] OR Reaven Syndrome[tw] OR syndrome X[tw] | [650,244](https://pubmed-ncbi-nlm-nih-gov.ezproxy.uct.ac.za/?term=Cardiometabolic+diseases%5Btw%5D+OR+Cardiometabolic+disease%5Btw%5D+OR+Cardiometabolic+syndrome%5Btw%5D+OR+Cardiometabolic+traits%5Btw%5D+OR+Cardiometabolic+risk+factors%5Btw%5D+OR+Cardiometabolic+factors%5Btw%5D+OR+cardiovascular%5Btw%5D+OR+Metabolic+Syndrome%5Btw%5D+OR+Metabolic+Syndrome+X%5BMeSH+Terms%5D+OR+Reaven+Syndrome%5Btw%5D+OR+syndrome+X%5Btw%5D&ac=no&sort=relevance) |
| 2 | Obesity[MeSH Terms] OR obesity[tw] OR obese[tw] OR overweight[tw] OR adiposity[tw] OR body mass index[tw] OR BMI[tw] OR Waist circumference[tw] OR waist [tw] OR hip circumference[tw] OR waist-to-hip ratio[tw] OR waist-to-height ratio[tw] | [594,043](https://pubmed-ncbi-nlm-nih-gov.ezproxy.uct.ac.za/?term=Obesity%5BMeSH+Terms%5D+OR+obesity%5Btw%5D+OR+obese%5Btw%5D+OR+overweight%5Btw%5D+OR+adiposity%5Btw%5D+OR+body+mass+index%5Btw%5D+OR+BMI%5Btw%5D+OR+Waist+circumference%5Btw%5D+OR+waist+%5Btw%5D+OR+hip+circumference%5Btw%5D+OR+waist-to-hip+ratio%5Btw%5D+OR+waist-to-height+ratio%5Btw%5D&ac=no&sort=relevance) |
| 3 | Hypertension[MeSH Terms] OR High blood pressure [tw] OR raised blood pressure[tw] OR Blood pressure[tw] OR systolic blood pressure[tw] OR diastolic blood pressure[tw] OR SBP[tw] OR DBP[tw] OR elevated blood pressure[tw] | [638,526](https://pubmed-ncbi-nlm-nih-gov.ezproxy.uct.ac.za/?term=Hypertension%5BMeSH+Terms%5D+OR+High+blood+pressure+%5Btw%5D+OR+raised+blood+pressure%5Btw%5D+OR+Blood+pressure%5Btw%5D+OR+systolic+blood+pressure%5Btw%5D+OR+diastolic+blood+pressure%5Btw%5D+OR+SBP%5Btw%5D+OR+DBP%5Btw%5D+OR+elevated+blood+pressure%5Btw%5D&ac=no&sort=relevance) |
| 4 | Diabetes mellitus[MeSH Terms] OR diabetes[tw] OR diabetes mellitus[tw] OR type 2 diabetes mellitus[tw] OR type 2 diabetes[tw] OR glucose[tw] OR hyperglycemia[tw] OR hyperglycaemia[tw] OR Glycated haemoglobin[tw] OR HbA1c[tw] OR Impaired fasting glucose[tw] OR Fasting glucose[tw] OR dysglycemia[tw] OR dysglycaemia[tw] OR Glycated hemoglobin[tw] | [1,120,662](https://pubmed-ncbi-nlm-nih-gov.ezproxy.uct.ac.za/?term=Diabetes+mellitus%5BMeSH+Terms%5D+OR+diabetes%5Btw%5D+OR+diabetes+mellitus%5Btw%5D+OR+type+2+diabetes+mellitus%5Btw%5D+OR+type+2+diabetes%5Btw%5D+OR+glucose%5Btw%5D+OR+hyperglycemia%5Btw%5D+OR+hyperglycaemia%5Btw%5D+OR+Glycated+haemoglobin%5Btw%5D+OR+HbA1c%5Btw%5D+OR+Impaired+fasting+glucose%5Btw%5D+OR+Fasting+glucose%5Btw%5D+OR+dysglycemia%5Btw%5D+OR+dysglycaemia%5Btw%5D+OR+Glycated+hemoglobin%5Btw%5D&ac=no&sort=relevance) |
| 5 | Dyslipidemia[MeSH Terms] OR dyslipidaemia[tw] OR dyslipidemia[tw] OR lipids[tw] OR lipid[tw] OR cholesterol[tw] OR hyperlipidemia[tw] OR hyperlipidaemia[tw] OR hypercholesterolemia[tw] OR hypertriglyceridemia[tw] OR triglyceride[tw] OR triglycerides[tw] OR total cholesterol[tw] OR HDL[tw] OR LDL[tw] OR TG[tw] OR TC[tw] OR VLDL[tw] OR hyperlipoproteinemia[tw] OR hyperlipidaemia[tw] OR Lipid disorder[tw] OR HDL cholesterol[tw] OR high-density lipoprotein cholesterol[tw] OR LDL cholesterol[tw] OR low-density lipoprotein cholesterol[tw] OR HDL-C[tw] OR LDL-C[tw] OR hyperlipidaemia[tw] | [996,715](https://pubmed-ncbi-nlm-nih-gov.ezproxy.uct.ac.za/?term=Dyslipidemia%5BMeSH+Terms%5D+OR+dyslipidaemia%5Btw%5D+OR+dyslipidemia%5Btw%5D+OR+lipids%5Btw%5D+OR+lipid%5Btw%5D+OR+cholesterol%5Btw%5D+OR+hyperlipidemia%5Btw%5D+OR+hyperlipidaemia%5Btw%5D+OR+hypercholesterolemia%5Btw%5D+OR+hypertriglyceridemia%5Btw%5D+OR+triglyceride%5Btw%5D+OR+triglycerides%5Btw%5D+OR+total+cholesterol%5Btw%5D+OR+HDL%5Btw%5D+OR+LDL%5Btw%5D+OR+TG%5Btw%5D+OR+TC%5Btw%5D+OR+VLDL%5Btw%5D+OR+hyperlipoproteinemia%5Btw%5D+OR+hyperlipidaemia%5Btw%5D+OR+Lipid+disorder%5Btw%5D+OR+HDL+cholesterol%5Btw%5D+OR+high-density+lipoprotein+cholesterol%5Btw%5D+OR+LDL+cholesterol%5Btw%5D+OR+low-density+lipoprotein+cholesterol%5Btw%5D+OR+HDL-C%5Btw%5D+OR+LDL-C%5Btw%5D+OR+hyperlipidaemia%5Btw%5D&ac=no&sort=relevance) |
| 6 | ART-naive[tw] OR ART naive[tw] OR untreated[tw] OR Antiretroviral therapy naive[tw] OR Non-ART[tw] OR Non-ART users[tw] OR ART-unexposed[tw] OR HAART-naive[tw] OR HAART naive[tw] OR HAART-untreated[tw] OR HAART unexposed[tw] OR ARV-unexposed[tw] OR treatment-naive[tw] OR naive[tw] | [268,099](https://pubmed-ncbi-nlm-nih-gov.ezproxy.uct.ac.za/?term=ART-naive%5Btw%5D+OR+ART+naive%5Btw%5D+OR+untreated%5Btw%5D+OR+Antiretroviral+therapy+naive%5Btw%5D+OR+Non-ART%5Btw%5D+OR+Non-ART+users%5Btw%5D+OR+ART-unexposed%5Btw%5D+OR+HAART-naive%5Btw%5D+OR+HAART+naive%5Btw%5D+OR+HAART-untreated%5Btw%5D+OR+HAART+unexposed%5Btw%5D+OR+ARV-unexposed%5Btw%5D+OR+treatment-naive%5Btw%5D+OR+naive%5Btw%5D&ac=no&sort=relevance) |
| 7 | HIV[MeSH Terms] OR HIV[tw] OR HIV/AIDS[tw] OR human immunodeficiency virus[tw] OR acquired immunodeficiency syndrome[tw] OR AIDS[tw] OR acquired immunodeficiency syndrome[MeSH Terms] | [481,421](https://pubmed-ncbi-nlm-nih-gov.ezproxy.uct.ac.za/?term=HIV%5BMeSH+Terms%5D+OR+HIV%5Btw%5D+OR+HIV%2FAIDS%5Btw%5D+OR+human+immunodeficiency+virus%5Btw%5D+OR+acquired+immunodeficiency+syndrome%5Btw%5D+OR+AIDS%5Btw%5D+OR+acquired+immunodeficiency+syndrome%5BMeSH+Terms%5D&ac=no&sort=relevance) |
| 8 | #1 OR #2 OR #3 OR #4 OR #5 | [3,119,907](https://pubmed-ncbi-nlm-nih-gov.ezproxy.uct.ac.za/?term=%231+OR+%232+OR+%233+OR+%234+OR+%235&ac=no&sort=relevance) |
| 9 | #6 AND #7 AND #8 | [1,572](https://pubmed-ncbi-nlm-nih-gov.ezproxy.uct.ac.za/?term=%236+AND+%237+AND+%238&ac=no&sort=relevance) |

**SCOPUS**

Search Date: 02/06/2022

| **Search** | **Search Terms** | **Hits** |
| --- | --- | --- |
| 1 | ( TITLE-ABS-KEY ( "cardiometabolic disease" )  OR  TITLE-ABS-KEY ( "cardiometabolic syndrome" )  OR  TITLE-ABS-KEY ( "cardiometabolic trait" )  OR  TITLE-ABS-KEY ( "cardiometabolic risk factors" )  OR  TITLE-ABS-KEY ( "cardiometabolic factors" )  OR  TITLE-ABS-KEY ( cardiovascular )  OR  TITLE-ABS-KEY ( "metabolic Syndrome" )  OR  TITLE-ABS-KEY ( "Reaven Syndrome" )  OR  TITLE-ABS-KEY ( "Syndrome X" ) ) | [1,064,775](https://www-scopus-com.ezproxy.uct.ac.za/search/history/results.uri?origin=searchhistory&shid=4) |
| 2 | ( TITLE-ABS-KEY ( obesity )  OR  TITLE-ABS-KEY ( obese )  OR  TITLE-ABS-KEY ( overweight )  OR  TITLE-ABS-KEY ( over-weight )  OR  TITLE-ABS-KEY ( adiposity )  OR  TITLE-ABS-KEY ( "body mass index" )  OR  TITLE-ABS-KEY ( bmi )  OR  TITLE-ABS-KEY ( "waist circumference" )  OR  TITLE-ABS-KEY ( "hip circumference" )  OR  TITLE-ABS-KEY ( "waist-to-hip ratio" )  OR  TITLE-ABS-KEY ( "waist-to-height ratio" ) ) | [738,497](https://www-scopus-com.ezproxy.uct.ac.za/search/history/results.uri?origin=searchhistory&shid=5) |
| 3 | ( TITLE-ABS-KEY ( hypertension )  OR  TITLE-ABS-KEY ( "High blood pressure" )  OR  TITLE-ABS-KEY ( "raised blood pressure" )  OR  TITLE-ABS-KEY ( "blood pressure" )  OR  TITLE-ABS-KEY ( "systolic blood pressure" )  OR  TITLE-ABS-KEY ( "diastolic blood pressure" )  OR  TITLE-ABS-KEY ( sbp )  OR  TITLE-ABS-KEY ( dbp )  OR  TITLE-ABS-KEY ( "elevated blood pressure" ) ) | [1,217,679](https://www-scopus-com.ezproxy.uct.ac.za/search/history/results.uri?origin=searchhistory&shid=6) |
| 4 | ( TITLE-ABS-KEY ( "Diabetes mellitus" )  OR  TITLE-ABS-KEY ( diabetes )  OR  TITLE-ABS-KEY ( "type 2 diabetes mellitus" )  OR  TITLE-ABS-KEY ( "type 2 diabetes" )  OR  TITLE-ABS-KEY ( glucose )  OR  TITLE-ABS-KEY ( hyperglycemia )  OR  TITLE-ABS-KEY ( "glycated haemoglobin" )  OR  TITLE-ABS-KEY ( hba1c )  OR  TITLE-ABS-KEY ( "impaired fasting glucose" )  OR  TITLE-ABS-KEY ( "fasting glucose" )  OR  TITLE-ABS-KEY ( dysglycemia )  OR  TITLE-ABS-KEY ( "glycated hemoglobin" ) ) | [1,620,699](https://www-scopus-com.ezproxy.uct.ac.za/search/history/results.uri?origin=searchhistory&shid=7) |
| 5 | ( TITLE-ABS-KEY ( dyslipidemia )  OR  TITLE-ABS-KEY ( lipid )  OR  TITLE-ABS-KEY ( cholesterol )  OR  TITLE-ABS-KEY ( hyperlipidemia )  OR  TITLE-ABS-KEY ( hypercholesterolemia )  OR  TITLE-ABS-KEY ( hypertriglyceridemia )  OR  TITLE-ABS-KEY ( triglyceride )  OR  TITLE-ABS-KEY ( total  AND cholesterol )  OR  TITLE-ABS-KEY ( hdl )  OR  TITLE-ABS-KEY ( ldl )  OR  TITLE-ABS-KEY ( tg )  OR  TITLE-ABS-KEY ( tc )  OR  TITLE-ABS-KEY ( vldl )  OR  TITLE-ABS-KEY ( hyperlipoproteinemia )  OR  TITLE-ABS-KEY ( hyperlipidemia )  OR  TITLE-ABS-KEY ( "lipid disorder" )  OR  TITLE-ABS-KEY ( "HDL cholesterol" )  OR  TITLE-ABS-KEY ( "high-density lipoprotein cholesterol" )  OR  TITLE-ABS-KEY ( "LDL cholesterol" )  OR  TITLE-ABS-KEY ( "low-density lipoprotein cholesterol" )  OR  TITLE-ABS-KEY ( hdl-c )  OR  TITLE-ABS-KEY ( ldl-c ) ) | [1,590,848](https://www-scopus-com.ezproxy.uct.ac.za/search/history/results.uri?origin=searchhistory&shid=8) |
| 6 | ( TITLE-ABS-KEY ( art-naive )  OR  TITLE-ABS-KEY ( "ART naïve" )  OR  TITLE-ABS-KEY ( untreated )  OR  TITLE-ABS-KEY ( "antiretroviral therapy naïve" )  OR  TITLE-ABS-KEY ( non-art )  OR  TITLE-ABS-KEY ( art-unexposed )  OR  TITLE-ABS-KEY ( haart-naive )  OR  TITLE-ABS-KEY ( "HAART naïve" )  OR  TITLE-ABS-KEY ( haart-untreated )  OR  TITLE-ABS-KEY ( "HAART unexposed" )  OR  TITLE-ABS-KEY ( arv-unexposed ) OR  TITLE-ABS-KEY ( naive ) OR  TITLE-ABS-KEY ( treatment-naive ) ) | [397,549](https://www-scopus-com.ezproxy.uct.ac.za/search/history/results.uri?origin=searchhistory&shid=9) |
| 7 | ( TITLE-ABS-KEY ( hiv )  OR  TITLE-ABS-KEY ( "HIV/AIDS" )  OR  TITLE-ABS-KEY ( aids )  OR  TITLE-ABS-KEY ( "human immunodeficiency virus" )  OR  TITLE-ABS-KEY ( "acquired immunodeficiency syndrome" )  OR  TITLE-ABS-KEY ( acquired  AND immunodeficiency  AND syndrome ) ) | [663,537](https://www-scopus-com.ezproxy.uct.ac.za/search/history/results.uri?origin=searchhistory&shid=10) |
| 8 | #1 OR #2 OR #3 OR #4 OR #5 | [4,665,933](https://www-scopus-com.ezproxy.uct.ac.za/search/history/results.uri?origin=searchhistory&shid=11) |
| 9 | #6 AND #7 AND #8 | [2,211](https://www-scopus-com.ezproxy.uct.ac.za/search/history/results.uri?origin=searchhistory&shid=12) |

**CINAHL via EBSCOhost**

Search Date: 02/06/2022

| **Search** | **Search Terms** | **Hits** |
| --- | --- | --- |
| S1 | (MH "Metabolic Syndrome X+") OR (MH "Cardiometabolic Risk Factors") OR (MH "Syndrome X") OR Cardiometabolic disease OR Cardiometabolic syndrome OR Cardiometabolic traits OR Cardiometabolic factors OR cardiovascular OR Metabolic Syndrome OR Reaven Syndrome OR syndrome X | 207,110 |
| S2 | (MH "Waist-Hip Ratio") OR (MH "Waist Circumference") OR (MH "Obesity+") OR (MH "Body Mass Index") OR Obesity OR obese OR overweight OR adiposity OR body mass index OR BMI OR Waist circumference OR hip circumference OR waist-to-hip ratio OR waist-to-height ratio | 233,152 |
| S3 | (MH "Hypertension+") OR (MH "Blood Pressure") OR (MH "Diastolic Pressure") OR (MH "Systolic Pressure") OR Hypertension OR High blood pressure OR raised blood pressure OR Blood pressure OR systolic blood pressure OR diastolic blood pressure OR SBP OR DBP OR elevated blood pressure | 185,716 |
| S4 | (MH "Diabetes Mellitus+") OR diabetes OR hyperglycemia OR Glycated haemoglobin OR HbA1c OR fasting glucose OR dysglycemia | 247,725 |
| S5 | (MH "Hyperlipidemia+") OR (MH "Lipids+") OR dyslipidemia OR lipid OR cholesterol OR hyperlipidemia OR hypercholesterolemia OR hypertriglyceridemia OR triglyceride OR total cholesterol OR hyperlipoproteinemia OR HDL cholesterol OR high-density lipoprotein cholesterol OR LDL cholesterol OR low-density lipoprotein cholesterol OR HDL-C OR LDL-C | 172,266 |
| S6 | Highly Active Antiretroviral Therapy naïve OR Antiretroviral therapy naive OR ART naive OR ART untreated OR Non-ART OR ART-unexposed OR HAART naive OR HAART-untreated OR HAART unexposed OR ARV-unexposed OR naive OR untreated OR treatment-naive | 34,811 |
| S7 | (MH "Acquired Immunodeficiency Syndrome") OR (MH "Human Immunodeficiency Virus+") OR HIV OR HIV/AIDS OR human immunodeficiency virus OR acquired immunodeficiency syndrome OR AIDS | 155,346 |
| S8 | S1 OR S2 OR S3 OR S4 OR S5 | 769,065 |
| S9 | S6 AND S7 AND S8 | 388 |
